# A thematic analysis of experiences of HIV risks among female sex workers in the Yunnan-Vietnam Chinese border region

**DOI:** 10.1186/s12905-020-01143-x

**Published:** 2021-01-06

**Authors:** Amanda Wilson, Yuan Yuan Wang, Runsen Chen, Ping Cen, Yuehui Wang, Xing Yao, Tang Wang, Shiyue Li, Hong Yan

**Affiliations:** 1 Division of Psychology, Faculty of Health and Life Sciences, De Montfort University, Leicester, UK; 2 Department of Psychiatry, University of Oxford, Oxford, UK; 3 Center for Disease Control and Prevention of Nanning, Nanning, China; 4 School of Health Sciences, Wuhan University, Wuhan, China; 5 Center for Disease Control and Prevention of Yuzhong District of Chongqing, Chongqing, China; 6 Center for Disease Control and Prevention of Wuhan, Wuhan, China

**Keywords:** Human immunodeficiency virus (HIV), Female sex workers (FSWs), Risk behavior, Yunnan province, China

## Abstract

**Background:**

The Yunnan province is located near the “Golden Triangle” border region between China, Myanmar and Thailand, which has the highest HIV/AIDS prevalence in China. Female sex workers (FSWs) in the Yunnan province are highly vulnerable to HIV infection. The objective of this study was to examine the experiences of FSWs in the Yunnan to better understand the risk of infection and the potential for transmission of HIV.

**Methods:**

Semi-structured interviews were conducted between May 2018 and June 2018 with 20 FSWs recruited in Hekou County, Yunnan Province, China. Thematic analysis was conducted to identify themes that highlighted increased exposure of FSWs to the risk of HIV infection and transmission.

**Results:**

The findings showed that FSWs’ primary source for HIV information was gynecologists, with few visiting the local HIV charity Red Ribbon. FSWs reported infrequent visits for check-ups with some seeing a gynecologist once a year. FSWs felt that the onus was on them to prevent STI/HIV infection by using a condom during sex, regardless of their ability to negotiate use. FSWs were also reluctant to see a gynecologists for treatment. Instead, they resorted to douching as a way of preventing HIV/STIs and treating vaginal health problems, such as leucorrhea. Most FSWs worked without the influence of alcohol and drugs. A small number of FSWs reported heroin addiction and injecting drug use.

**Conclusion:**

The findings suggest a need for innovative HIV prevention strategies among FSWs and their clients in the Chinese border region. Governmental agencies should continue to implement practical strategies in terms of HIV prevention education and condom use through tailored interventions that are localized. Such strategies should include localized tailored interventions that dispel myths about douching as a method of HIV/STI prevention and incorporate a mobile outreach approach, similar to the ‘roadside restaurant’ outreach that has been successful in rural China.

## Background

### Introduction

Globally, female sex workers (FSWs) are a highly vulnerable population to the human immunodeficiency virus (HIV). FSWs are both at risk of transmission from HIV positive clients and HIV positive FSWs can transmit the virus to the general population through sex work. A previous meta-analysis published in 2012, covering 50 countries, reported that the overall global HIV prevalence among FSWs was 11.8% [1]. China’s sex work industry is also increasing rapidly for FSWs due to an upward trend in economic prosperity and the growing disproportionate gender ratio where there are more males than females in the total Chinese population [2]. Prevention initiatives in China promote male condoms; however, studies among FSWs show that condom use varies across settings, which suggests a need for further research to understand trends in HIV risk to manage and prevent transmission through condom use [3]. Zeng et al. [4] suggest that both sex workers and clients tend to have low awareness and understanding of HIV transmission and prevention strategies. In a systematic literature review of interventions for sexually transmitted infections (STIs)/HIV among FSWs, condom use and education interventions were common; however, high rates of STIs/HIV continued despite the interventions, leading the researchers to call for more diverse interventions that are rigorous, biomedical and structurally innovative to reduce the risk of HIV/STI infection [5]. Sex work is illegal in China, which makes FSWs a hard-to-reach population for public health interventions, including STI/HIV prevention. Research on FSWs’ male clients show conflicting findings and the prevalence of sexually transmitted infections (STIs) /HIV remains high (6, 7), providing little insight into how male clients’ use of condoms mediate risk of HIV and STIs. There have been some reports in China of FSWs contracting HIV, through the selling of blood/plasma; however, the majority of risks come from condom less heterosexual behavior and injecting drug use (IDU) [8]. Outside of FSWs who inject or use drugs the risk of HIV transmission varies depending on the environment the FSW works in. Sex workers are a diverse group working in a wide range of different contexts [9]. Many HIV prevention programs for sex workers lack a sufficient understanding of the epidemiological transmission dynamics and the consideration of geographic and population heterogeneity [10]. Effective HIV prevention for sex workers should also be tailored according to the environment where FSWs work (11, 12).

Although many studies have focused on the FSWs located around the Chinese border region [2], there is still limited in-depth understanding of the HIV burden and risk for these women. There is overall limited research to explain poor health promotion for sexual and reproductive health among the FSWs [13]. A qualitative study conducted in Yunnan in 2003 found lower risk of HIV transmission among younger than among older FSWs, and limited condom use among FSWs despite availability of the method [14]. The HIV related burden among FSWs in the Yunnan still requires increasing research attention as the province continues to have the highest HIV prevalence in China [15]. We conducted a qualitative study in Yunnan to gain an in-depth understanding of the unique experiences of FSWs regarding the risks of HIV infection. The research question was, how do Chinese female sex workers in the Yunnan border of China and Vietnam experience risks of HIV?

### Study context

Yunnan is located near the “Golden Triangle” of the border region between Thailand, Myanmar, and China, and has the highest HIV/Acquired Immune Deficiency Syndrome (AIDS) prevalence in China (16, 17). The Yunnan province is indeed considered an epicentre of HIV in China, with epidemiologists believing that the outbreak of HIV in the province is the root cause of the spread of HIV throughout China [18]. Researchers have found a diversity of HIV-1 genetics with six genotypes in the province, which is unlike any other region in China, and which is a further indication of the spread of HIV from the Yunnan to mainland China [19]. HIV prevalence in the region is considered to have reached epidemic proportions, with heterosexual intercourse being the primary reason for transmission and transmission from people who inject drugs (PWID) is on the decline [20]. The literature shows inconsistent patterns of HIV prevalence among those engaging in heterosexual intercourse. One longitudinal study showed that from 2006 to 2009 HIV prevalence remained stable [21], particularly among PWIDs. However, it was stable at a higher rate than the rest of China and there was still an unmet need for interventions to reduce the risk of infection, including Needle Syringe Exchange programs and Methadone Maintenance Therapy [22]. In another longitudinal study spanning 10 years, researchers reported that in 2006 the main route of HIV transmission changed from PWIDs to sexual transmission, which rose from 30.6 to 58.4% between 2006 and 2010 [23]; this change has been predicted to continue using mathematically based epidemic models [24].

HIV transmission in China was originally associated with PWIDs in the 125 counties of the Yunnan, with the increasing trend of transmission among FSWs starting in 2005 [25]. In the first provincial study of the Yunnan, FSWs had the highest percentage of new HIV cases (16.4%), which was above the global average of 11.8%; however, the estimates of new HIV cases in the region could under-estimate incidence among heterosexual men, many of whom do not test regularly and do not know their HIV status [26]. A study by Chen et al. [27], among FSWs from 1989 to 2007, showed that HIV prevalence increased from 0% to 4%, which is above the national average, but below the global average. The same study found that HIV prevalence among FSWs in China started rising from 2007 while heterosexual transmission increased from 37% to 66% by 2014 [27]. In contrast, the rate of HIV transmission in Zhuang Autonomous Region located along the Yunnan increased from 90% in 2012 to 93% in 2014 [27], showing that prevalence in the Yunnan was increasing at a higher rate than other provinces. Those who engage in sex work in temporary rented accommodation, or beauty salons are at a higher risk than those who operate from karaoke clubs, night clubs, saunas and hotel environments [28]. Several of the other known risks in the Yunnan include unprotected sexual intercourse, lack of support services, high STIs, and substance use.

Male clients often visit workers in the Yunnan for what is called a ‘fast snack’ where there is quick rigorous vaginal sex without lubricant, resulting in a higher likelihood of lesions and bleeding during sex, which also increases the risk of contracting HIV [29]. Male clients act as transmission channels for HIV to the general population [30], FSWs, or across borders, with research showing that truck drivers who come into the region have the greatest risk of spreading the virus both at home and abroad [2]. Male clients of FSWs in the Yunnan report having numerous sexual partners and using condoms less frequently [31]. As far as engaging with services is concerned, Yunnan FSWs find that services take the same monotonous approach that has several challenges, including lack of enough publicity, access barriers, stigma, and inadequate services [32]. Previous research has also pointed out that it is difficult for government agencies in the Yunnan to provide services to sex workers due to lack of mutual trust [19]. Only 28.4% of Yunnan FSWs report being involved in intervention programs that aim to reduce HIV, which is an indication of the need to strengthen interventions to reach FSWs [33]. Despite increased awareness about HIV and condoms promotion, few FSWs in Yunnan return to know the results when they are tested for HIV, which highlights a need for point of care access testing that provides results on the same day [34]. Furthermore, 40% of Yunnan FSWs choose to buy their medicines themselves without visiting a clinic or general practitioner (GP), which is an indication of the need to improve health-seeking behaviors and healthcare systems [35].

FSWs in Yunnan are not only at an increased risk of HIV infection but STIs as well, which further increases the risk of contracting HIV [36]. STIs linked to increased risk of HIV infection in the region include chlamydia and gonorrhoea, herpes simplex virus-2 (HSV-2), trichomonas vaginalis and genital ulcers, syphilis, genital warts, and human pegivirus 2 [37–45]. Other female genital tract problems prevalent in the region include, leukorrhagia, vaginitis, and genital hyperplasia [46], while Hepatitis B and C are additional risks associated with the nature of sex work due to the potential for mixing of fluids and blood [47]. Mathematical modelling suggests that prevention measures could control new HIV infections among both FSWs and male clients [48]. However, research shows that the implementation of effective measures is yet to be realized.

There is a known relationship in the Yunnan between FSWs, injecting drugs, and having other STIs, all of which increase the risk of HIV transmission [49]. In addition, the risk of infection is high among those with low levels of education, minority groups, those who are older, and new injecting drug users [50]. Evidence shows that FSWs who are IDUs have a higher risk of contracting HSV-2, other STIs and HIV because of susceptibility associated with injections and being in the profession for long compared with those who do not use drugs [51]. Evidence further shows an increased risk of HIV infection among FSWs in Yunnan who use drugs regardless of the method of administration [52]. However, heterosexual contact remains the most predominant mode of HIV transmission among FSWs in Yunnan although injecting drugs use and alcohol are still considered high risk factors [26]. Research has shown that 40% of HIV infected individuals in the Yunnan abused alcohol (mostly Chinese white wine that is high in ethanol) in the month prior to diagnosis, with only 8% using a condom when having sex after taking alcohol [53]. However, male clients in the region who inject drugs in the Yunnan and do not use condoms are at a high risk of transmitting infections to FSWs and the general population [54] and of transmitting HIV to the FSWs [55].

#### Methods

### Settings and subjects

Semi-structured interviews were conducted between May, 2018 and June, 2018 with 20 Chinese FSWs recruited in Hekou County, Yunnan province, China. Participants were recruited trough the local Chinese Center for Disease Control and Prevention (CDC), with staff contacting ‘Madams’ (elderly experienced sex workers^56^) who then contacted the sex workers. The sex workers who came forward were both read an information sheet and provided a copy emphasizing voluntariness of participation, freedom to withdraw from the study at any time, and what to do if one had questions. Eligibility criteria for participants were: (1) age 18 years or above; (2) female sex workers, who self-reported providing sex in exchange for money or material gains within the last 3 months, and (3) ability to communicate with the researchers and provide oral informed consent to ensure they wanted to participate before the interview commenced. The study protocol was approved by the Wuhan University Research Ethics committee. Face-to-face interviews were conducted by trained researchers from Wuhan University. In total 20 Chinese FSWs were interviewed for this study. The demographic information of female sex workers who participated in the study is presented in Table 1. The sex workers had low income and were living in poverty, which made them engaged in both indoor and outdoor sex work in an effort to obtain enough money to pay their bills. Most participants were between 37 and 40 years old, with the youngest being in her late 20 s while the oldest was in her 50 s and although four did not provide their ages, they were roughly between 35 and 50 years old. Ten participants did not know their HIV status, six reported that they were HIV-negative while four indicated that they were HIV-positive. Eight women reported that they took alcohol but not necessarily when working, three indicated that they used drugs specifically injected heroin, while nine did not take alcohol or use drugs.

**Table 1:** Demographic information of participants

Participant number	HIV status	Age range	Drink or drugs	Friend or relative present
1	Positive	40–44	Drink occasionally, no drugs	No
2	Unknown	40–44	Drink occasionally, no drugs	No
3	Unknown	50–54	No drinking, no drugs	No
4	Unknown	Prefer not to respond	No drinking, no drugs	No
5	Unknown	25–29	Drinking ½ dozen beers, no drugs	Sister
6	Positive	35–39	No drinking, Injecting drug user (heroin), methadone user, self–identified as drug addict	No
7	Unknown	30–34	No drink, no drugs	No
8	Positive	30–34	Couple glasses of wine, no drugs	No
9	Negative	50–54	No drink, no drugs	No
10	Positive	30–34	No drink, Injecting drug user (heroin), methadone user	No
11	Unkown	35–39	No drink, injecting drug user (heroin), Outpatient rehabilitation Centre,	No
12	Negative	30–34	No drink, no drugs	No
13	Unknown	40–44	Occasionally wine identified as non-drinker,	No
14	Unknown	Prefer not to respond	Drink with familiar client, No drink with the unfamiliar client	No
15	Negative	Prefer not to respond	Drink a little beer, no drugs	No
16	Negative	Prefer not to respond	No drink, no drugs	No
17	Unkown	50–54	Sometimes a little wine, no drugs	No
18	Negative	50–54	No drink, no drugs	No
19	Unknown	40–44	Use to drink before married, no drugs	No
20	Unkown	35–39	No drink, no drugs	No

### Data collection procedures

Two interviewers collected the data, with one interviewer present in each interview. The setting for the interviews was the workplace of the FSW if they had an indoor place of work, with some FSWs living in areas where they worked. This was done to create an environment that allowed the sex workers to speak freely. The sex workers were interviewed once and could have a friend or relative present if they chose to. Only one of the sex workers had her sister present while the other FSWs said they did not want to have a friend or relative present during the interview. Having her sister present could have biased the responses in the interview, but the comfort of the FSW was considered most important. Interviews lasted on average 45 min. The interviews were audio-recorded with consent of FSWs. Interviews were conducted in Chinese, transcribed verbatim, and translated into English, with a standard of checks completed that included translation and back translation [57]. A semi-structured topic schedule was used to capture the FSWs’ experiences; the topic schedule for this study is provided as Additional File 1. The main domains included: Current Situation, Reasons for Sex Work, Border Flow Pattern, and HIV Transmission. The research was inductive with themes grounded in data, which means that the final themes could have little resemblance to the series of interview questions^58^. As such only three of the domains, Current Situation, Reasons for Sex Work, and HIV Transmission, were reflected in the final themes.

### Data analysis

The analysis was conducted manually using a psychological form of thematic analysis [55]. It was inductive, being driven by the data, and themes were also semantic, meaning they represented a surface level interpretation of the data, which fits with research that is exploratory. Without substantial previous qualitative research to guide the study, Thematic Analysis, a method that reflects reality at the surface level and has theoretical flexibility, was appropriate. The analysis was guided by the six stages of thematic analysis proposed by Braun and Clarke [58]. In stage one the lead researcher gained familiarity with the data by reading the (20) interviews a series of times, the interviewer then continued to actively re-read the interviews to identify patterns in the data. Notes were taken during stage 1 for ideas that could inform the initial codes. Initial ‘data driven’ coding was undertaken in stage 2 involving in vivo coding (e.g. condoms, checkup, AIDS, drugs, alcohol) and superordinate coding (e.g. drug and alcohol combined). This was done without fitting codes within a pre-existing format but forming revised codes from the data that allowed for data to be grouped in a meaningful way. The final coding was then used to construct the themes, and identify patterns in the data, completing stage 3. This involved grouping codes underneath similar themes, which was done using excel with each theme and its codes having its own sheet. The lead researcher used this format to visually display all data for each theme, and to sort and organize the information. Stage 4 involved revising the themes to best convey the information, with considerations to external and internal homogeneity. Extracts that best represented specific themes were also identified at this stage. It was also at this stage that investigator triangulation was undertaken, where two other researchers reviewed the themes for concurrence, with disagreements being resolved in consultation with the lead researcher [59]. The revised themes were given final names during stage 5, with the extracts being placed under the relevant themes and the narrative of what was interesting and why was written. In the final stage the results were written into a report, with extracts representing the majority of the sex workers’ views. Negative case studies, or narratives that were not present in all the interviews, are presented as appropriate around condomless sex, due to the high risk of HIV transmission, and drug use, as not all FSWs used drugs, which concluded stage 6 of the steps of thematic analysis.

## Results

There were four themes actively interpreted from the data by the researchers who identified the patterns and selected the themes relevant for answering the research question. They included: *Infrequent Access to Informational Support for HIV/AIDS*, *Personal Accountability for Using Condoms to Prevent STIs*, *Gynecological Problems Commonly Self-Treated by Douching*, and *Working Without Being Intoxicated*. The themes are presented below, with extracts to support how the theme has been constructed.

### Infrequent access to informational support for HIV/AIDS

The narratives showed that the FSWs had limited access to informational support for HIV/AIDS because of inability to attend regular check-ups by gynecologists who were their primary source of information, and limited engagement with the HIV/AIDS charity operating in the study setting, the Red Ribbon. Relying only on gynecologists limited the women’s opportunities for accessing comprehensive HIV/AIDS information due to lack of regular contacts with the providers occasioned by lack of time and opportunity costs of missing out on clients. In addition, the workers did not know much about AIDS despite the presence of Red Ribbon, a non-profit that provides integrated care and support for those living with HIV, which was an indication of limited contact with the program.

Female Sex Worker 6: I don't know. I didn’t know about the AIDS thing before.

Interviewer: Hadn’t the red ribbon been there [in the community] to discuss this information?

Female Sex Worker 6: I haven't been there [to the red ribbon charity for HIV]. I have only heard of syphilis before. I had never heard of AIDS. The first time I heard was when I had done an examination [with a gynecologist who diagnosed me]. For the Miss [Madam], I checked it out [my HIV status]. (drawing the seventh cigarette).

-Age Range: 35–39, HIV Status: Positive

When asked if she was informed about AIDS, Female Sex Worker 6 stated she did not know, saying this twice for emphasis. Her knowledge was limited to one STI, syphilis; she had just completed her first gynecological exam where she learned about AIDS. The limited information the workers had could be because gynecologists did not visit regularly and the women believed they would know if they had HIV/AIDS without a test:

Interviewer: What do you think about AIDS?

Female Sex Worker 15: We are going to the red ribbon [charity’s gynecological service]. They [the gynecologists] are coming from time to time [and providing services]. It’s just a physical examination, the wall will be written [the writing is on the wall, or they know if they are positive], we all know.

-Age Range: Prefer Not to Respond, HIV Status: Unknown

The worker reported that gynecologists did not come regularly for outreach services, referring to her visits as ‘coming from time to time’. She uses the word ‘just’ to imply that the physical exam provided is not enough and that the workers know if there is a physical problem, ‘the wall will be written’. She also implied that she was informed about AIDS, aware that one may be asymptomatic when infected. The Red Ribbon program operated effectively when it was just established, but at the time of the study, FSWs had infrequent contacts with the charity:

Female Sex Worker 5: When we used to collect blood [for HIV testing] we told them [Red Ribbon] not to go [not to leave]. At that time, the doctor went directly to each dance hall. At that time, we were all relatively fixed [sex workers were regularly tested]. Every dance hall had a good business [because there was regular testing]…

Interviewer: Then how did you get to the doctor?

Female Sex Worker 5: When the Red Ribbon was just established, when my sister used to play [, do sex work], I told my sister that when I was fine [tested negative], that I went to chat [at the Red Ribbon charity] and played cards [gambled]. I followed up [for my test results], and then recruited staff. I had been doing it for six or seven years, but I haven’t done it for a few years [HIV testing]. I said [to the charity] that I have to go home [moved away]…

Interviewer: Do you usually go out for outreach [with the Red Ribbon]?

Female Sex Worker 5: We had outreach here [from Red Ribbon]. I was in charge of this office. Every place had outreach and we did not need to worry [about testing for HIV]. In the past, we also had to go out to sensitize communities [on HIV]. Before, we had set up a villa. When we were in the Red Ribbon, we had to come over here [to sensitize the community] frequently. Many people lived together. There were more than ten people in the middle of the road. They were brought together, sensitization occurred at home.

-Age Range: 25–29, HIV Status: Negative

Red Ribbon was a program that Female Sex Worker 5 felt was important, she did not want them ‘to go’. The key to the success of the program was going to the dance halls where the women worked. Female Sex Worker 5 was involved with Red Ribbon as she was doing indoor work at the time, had free time, and lived in the locality. The worker did not have a charity to engage with when she moved back home and was unable to find a place for HIV testing, unlike in the past when she tested for ‘six or seven years’. She was formerly in charge of the outreach office for Red Ribbon and places of business ‘did not need us to worry’, indicating that when outreach was effective, the workers did not worry about HIV risks. She uses “we statements” to show that it takes a team of people to be effective. Other important aspects of the program included visiting halls frequently and sensitizing the community about AIDS. She indicated that sensitization occurred in the community, ‘ten people in the middle of the road. They were brought together… at home’.

### Personal accountability for using condoms to prevent STIs

The FSWs’ narratives indicated that they were responsible for condom use and that they were responsible for checking a man’s penis to see if he had an STI. They reported that the majority of their clients used condoms and if they had condomless sex they received a higher pay. Although the FSWs experienced barriers to accessing informational support for HIV/AIDS, they understood that by using condoms they were protected from STIs. The workers preferred clients to use condoms, though the reality was that if they needed money they would charge more for not using one. The few who knew they were HIV positive insisted that clients use condoms; however, a majority of the participants did not know their status. Regardless of the clients age, most agreed to use a condom:

Interviewer: Who asks to use a condom?

Female Sex Worker 12: I ask for it, I ask for it (laughing), I don't need to set it up [it is pre arragned] (laughing)

Interviewer: Do the 50–60-year-old males bring a condom ?

Female Sex Worker 12: I can't do it without a condom. If they don't wear it, I don't agree (shakes her head).

Interviewer: If they don't agree, then they are not your guest [client], but are there [a] few [who do not use a condom]?

Female Sex Worker 12: No, there is not, I don’t want this money [from condomless sex]…Some don’t have a condom. I don’t want to [have sex without a condom]. If he doesn’t agree [to use a condom], he’s gone.

-Age Range: 35–39, HIV Status: Negative

The sex worker used ‘I’ repeatedly to position herself as responsible when it came to condom use, by, for example, using the words “I don’t’ and ‘I can’t’. She negotiates in advance to use condoms with both younger and older clients. She reported that she turned away clients who could not use a condom by stating that, ‘I don’t want this money’, ‘If he doesn’t agree, he’s gone”.The FSWs also checked the men for STIs:

Interviewer: Are the friends[clients] around you wearing them [condoms]?

Female Sex Worker 18: Some [condoms] are worn, young people wear it [condoms], that is, if they are old, that is, there are many people wearing condoms, less are not wearing [condoms].

Interviewer: Are you afraid that some guests [clients] have something wrong [e.g. STI]?

Female Sex Worker 18: Well, we have to look [at his penis] first.

Interviewer: Can you usually see this [, STIs]?

Female Sex Worker 18: Yes.

Interviewer: Is the guest angry [if you check his penis]?

Female Sex Worker 18: He is angry and not angry [if I check his penis], his body [being healthy] is the most important thing.

-Age Rnage: 50–54, HIV Status: Negative

Female Sex Worker 18 reinforced the narrative that not using a condom occured ‘less’ and is not related to age. To determine if the client is free from STIs she first has a look at his penis, and she did not care if this angers the client. She positioned herself as looking out for the client’s health by stating that ‘his body is the most important thing’ but fails to acknowledge the benefit to her own body. However, while the FSWs reported a desire for condom use, in reality when condoms were not used, the price they could charge was raised:

Interviewer: How much is their actual use [of condoms]?

Female Sex Worker 7: There are many [who would use a condom] (laughs)

Interviewer: Do you not need [to use a condom]?

Female Sex Worker 7: No, use [of condoms], Yes, yes, um

Interviewer: None of the them [use a condoms], the ones where you work inside [do they use a condom]? Actually, do men not like to use it [condoms]?

Female Sex Worker 7: If it [a condom] is introduced, most of them [clients] are older. If you are afraid of getting sick, you must use it [a condom].

Interviewer: Does the guest ever use their old age as an excuse [not to use a condom]?

Female Sex Worker 7: That's there too.

Interviewer: Is there a situation where they don’t want to use [a condom]?

Female Sex Worker 7: Yes, there is

Interviewer: So is there any use in this situation?

Female Sex Worker 7: There is no use [of a condom], [and I] add money [to not use a condom]

Interviewer: So you want to add money if you don't need it [a condom with a client]?

Female Sex Worker 7: I want to add one hundred dollars [to not use a condom].

-Ag Rangee: 30–34, HIV Status: Unknown

The worker admited that sometimes male clients do not use condoms; however, this is inconsistent with the other workers’ responses as she suggested that, ‘there are many’. She agrees with the other FSWs that, if there is a concern with the clients’ health, the workers ‘must use it’; again the onus of preventing STIs is on the worker, ‘if you are afraid’. This concern around not using a condom was more with younger men, while ‘older’ men were more likely to suggest that they use a condom, indicating that the likelihood of male responsibility for condom use may increase with age. If a client did not want to use a condom the price for sex increased; she would add ‘one hundred dollars’, a large sum and profit, particlarly given the poverty these women lived in.

### Gynecological problems commonly self-treated by douching

The FSWs were unlikely to seek medical care if they developed vaginal symptoms and instead performed their own health checks. Poor vaginal health was commonly reported; vaginal problems could have been due to the FSWs’ douching both after sex and to treat any symptoms they experienced. If the workers did get a gynecological exam it was only once a year, even though they regularly engaged in sex work:

Interviewer: Then how are you going to re-examine [your vagina frequently]?

Female Sex Worker 16: I am not part of the Red Ribbon [charity]. I will have a physical [gynecological] examination every year, because we are going back outside [to do street sex work], no matter what we do now. We do cancer screening every year.

Interviewer: Is it quite good?

Female Sex Worker 16: Yes, [the service] it's normal.

-Age Range: Prefer Not to Respond, HIV Status: Negative

The worker explains that she is not part of the Red Ribbon HIV/AIDS program and that she is HIV negative. By not being part of the program, she goes for a physical exam and cancer screening only once a year. She implies that women who do street work are stuck on the trade by stating that, ‘we are going back outside, no matter what’. She felt that the gynecological services were of average quality by stating that they were just ‘normal’. Without bespoke gynecological services, there was limited formal support to improve vaginal health. The workers commonly reported ‘itchy’ vaginas and leukorrhea as a result:

Interviewer: Have you checked [your vaginal health with a gynecologist] in the past two years?

Female Sex Worker 2: Not checked.

Interviewer: Is there self inspection [of your vaginal health]?

Female Sex Worker 2: Well, I just check it [my vagina] out myself…

Interviewer: Is it usually uncomfortable [your vagina]? Is it uncomfortable below?

Female Sex Worker 2: I just had a self check [of my vagina today], it is a little uncomfortable below.

Interviewer: How uncomfortable?

Female Sex Worker 2: Itchy…

Interviewer: It's just itchy, nothing else?

Female Sex Worker 2: Well, [my vagina is] itchy, today is my first time to check.

Interviewer: Yeah, do you have to check it [your vagina] often?

Female Sex Worker 2: Do more self- inspection [than gynecological].

Interviewer: Do you have a regular doctor? The doctors who control the [sexually transmitted] disease are they quite good?

Female Sex Worker 2: I don't know…

Interviewer: Then, are you going to continue working and suffering, have you thought about it [what you are going to do about your vaginal itch]?

Female Sex Worker 2: Don't want to suffer [with vaginal itch]

Interviewer: Don't want to suffer? Why don't you want to go back [to visit a gynecologist]?

Female Sex Worker 2: I don't want to go back [to the gynecologist]. Hey, I don't want to work so hard in this place, I want to change places [to improve my vaginal health].

-Age Range: 40–44, HIV Status: Unknown

This worker was in her 40 s but did not have a regular doctor. She reported that she had just completed a self check-up and her vagina felt ‘uncomfortable below’, using the word ‘itchy’ twice for emphasis. While she wanted to acess vaginal health and treat her problem by stating that, ‘don’t want to suffer’, she did not want to go back to the gynecologist. She wanted to ‘change places’ of work where she did not need to work as hard, as she associated her poor vaginal health to working hard (seeing many clients in less than ideal conditions) in her current place of work. Sex workers who participated in the study had other gynecological problems, including fibroids and leucorrhea. They not only did self-examinations of their vaginas but also engaged in douching regularly:

Interviewer: Have you ever had any gynecological diseases?

Female Sex Worker 13: My uterus seems to be a little enlarged, I have uterine fibroids

Interviewer: Is it cervical hypertrophy?

Female Sex Worker 13: A little hypertrophy of the cervix, but there are uterine fibroids

Interviewer: Is it engorged?

Female Sex Worker 13: No

Interviewer: Abnormal leucorrhea?

Female Sex Worker 13: That is normal [I do have leucorrhea].

Interviewer: Is there an itch [with your vagina]?

Female Sex Worker 13: Occasionally [I have vaginal itch], I use Fu Yan Jie wash [to treat it]

Interviewer: Is that how to clean [ your vagina] after doing the work with the guests?

Female Sex Worker 13: That is right, it [my vagina] is generally washed with water too. After washing, I don’t know what it is, but it [my vagina] is sometimes oily.

Interviewer: What if he ejaculates inside? [Do you] Wash it [your vagina] inside [if he ejaculates inside]?

Female Sex Worker 13: Washing [I wash my vagina if he ejaculates inside]

-Age Range: 40–44, HIV Status: Unknown

Female Sex Worker 13 had ‘uterine fibroids’ which made her cervix ‘enlarged’ and she had ‘a little hypertrophy’, which could make sex work uncomfortable. She believed that having any leucorrhea was normal. If this occurred and she felt an itch she used a douche ‘Fu Yan Jie wash” to treat her vagina herself. She talked about good practice after having a client, that she could wash, and douche, afterwards. This perpetuates the myth that douching removes any risk of HIV/STIs infection.

### Working without being intoxicated

The FSWs explained that they used alcohol in moderation while they were working; this occurred for several reasons such as safety, avoiding loss of money, and minimizing arguments with other women. Drug use was rarely spoken of, only a couple women reported heroin use. Few sex workers reported addiction problems; the majority said they did not drink to excess or use drugs when working which shows that the FSWs practiced professionalism. Sex workers are encouraged to reduce risk, or ‘practice professionalism’, to remain in control during the work (whether controlling the level of penetration, or being able to escape a violent client), which was not possible if they are under the influence of drugs or alcohol. Several extracts supported an overall level of professional work mentality by FSWs:

Interviewer: Drinking?

Female Sex Worker 11: Don't drink, because I don't know if it is true, that is, drinking is in conflict with this [sex work].

-Age Range: 35–39, HIV Status: Unknown

Interviewer: Is the amount of alcohol good?

Female Sex Worker 16: I don't drink alcohol. I still choose to smoke two cigarettes. They [clients] drink two or three cups at a time, three or four cups, four or five cups. I don't advise you to drink here. Some places are very expensive.

-Age Range: Prefer Not to Respond, HIV Status: Negative

Interviewer: How much is smoking and drinking seen in the guests [clients]?

Female Sex Worker 20: Not much. I don't do anything [any sex work] when I drink alcohol. I don't know, if a regular customer calls me, because I like this person to be cheerful [I drink a little], some drink alcohol is fine [when doing sex work], but too much [alcohol for you and the client] and you are not welcome to talk and they can get ugly, I don't like it, so I do not drink the wine and do not do it [when out with clients I do not know]. There are a lot of sisters [fellow sex workers] who drink, and there is a lot of quarrels after drinking. I don't like to quarrel with people.

-Age Range: 35–39, HIV Status: Unknown

Female Sex Worker 11 identified drinking as being ‘in conflict with this’, suggesting that women should not drink when working, a view that was also held by Female Sex Worker 16 who said that, ‘I don’t advise you to drink here’. Those who took alcohol, like Female Sex Worker 20 above, kept the drinking to a minimal in order to appease their clients. Some of the workers chose not to drink due to cost, while others felt it was only safe to drink with a regular client they knew and would not drink with a new client. Female Sex Worker 16 only saw regular clients during or after they had been drinking, but she was put off by the ‘wine’ and did not like it when the client was drunk to a point she could not talk and he became ‘ugly’ or mean. They also avoided drinking to stay out of trouble; women could ‘quarrel’ after too much drinking, which was unwanted. The same applied to drugs, as most women did not speak of drug use:

Interviewer: How much drug was used 10 years ago?

Female Sex Worker 19: Well, it seemed to be small

Interviewer: Have you seen drug abuse?

Female Sex Worker 19: No, I have never seen it. I have never seen any drug abuse.

-Age Range: 40–44, HIV Status: Unknown

The sex worker repeatedly said, ‘I have never seen’ to emphasize that drug abuse was not common among the working women. It was, however, not clear from the narratives whether this was the case or the participant was giving socially desirable responses. If there was no addiction to drugs, participants still engaged in high-risk behaviors:

Interviewer: How do you prepare before going out?

Female Sex Worker 10: Use the needle [inject heroin] and eat well. It [the high] can be maintained until dawn.

Interviewer: How big is the amount of heroin used?

Female Sex Worker 10: One or two or three packets [of heroin], see if the purity is good, two can do, three packets can be used if poor, anyway, I figured that out when I went to stay overnight and also needed to support it[the addiction]…You can share the needle water [when injecting]. Everyone uses a new needle. We only want to use it [the syringe] once. We used to wash the needle [so we could re-use it].

Interviewer: Do you wash it together?

Female Sex Worker 10: I have washed it [cleaned and re-used a syringe]. Now I have changed some needles over at the Red Ribbon for new [used the needle exchange]. We can also buy it [new syringes] with money.

Interviewer: How did you get infected?

Female Sex Worker 10: I am [HIV positive], my husband. He didn't tell me [he was HIV positive] when I was with him. I don't know [when he became positive]. I didn't know when I got married. I had lived for so long [without getting HIV]; I had lived [as HIV negative] for a year or two when I got married, I hadn’t got it yet. I had to check for it [test for HIV] when I got married. He has it [now]. I didn't have it [HIV]. I only found out last year [I was HIV positive]. I was married for the past five years without [testing positive].

-Age: Range: 30–34, HIV Status: Positive

Female Sex worker 10 was an injecting drug user who injected heroin and had HIV. To avoid withdrawal, she needed up to three packets of heroin depending on whether she was spending the night with a client, something that she learnt over time, which indicates that she used the drug frequently when working. She engaged in risky behavior by sharing water and washing syringes, which were then re-used. She did occasionally engage in good practice though by not sharing needles and trying to use the Red Ribbon needle exchange program or by buying clean needles. She acquired HIV from her husband. Her husband did not tell her that he was HIV-positive; she only found out a year before the interview and could have been infected sexually or through poor injecting practices.

## Discussion

This study explored the HIV risks among FSWs in Yunnan, and provides an in-depth understanding of the experiences of FSWs. The narratives revealed intersecting influences of sex work and identified context specific information that can inform interventions to reduce HIV/STI risk among FSWs in the region. The FSWs worked in outdoor environments. The literature relates being an indoor worker to low risk of HIV [49] but in this study the same women reported working both outdoors and indoors, which put them at a high risk of HIV and STIs. Future research should account for the cross-over between the different environments of outdoor and indoor work of FSWs instead of considering these groups as two distinct and different categories of risk. The study highlights the importance of promoting HIV prevention strategies for FSWs and their clients, suggesting the need for further outreach activities. Moreover, the results showed that FSWs try to consciously protect themselves from risks of poor vaginal health, contraction of HIV/STIs, and personal safety, when they could.

Low awareness levels are a risk factor for HIV transmission and increase the likelihood of non-use of condoms among FSWs whose male partners insist on sex without a condom [60]. It is challenging for public health professionals and clinical practitioners to promote HIV-related knowledge among the sex worker population [19]. A 9 year longitudinal study in the Yunnan Province also found that there was limited follow-up with health professionals due to the nature of sex work [64]. Due to stigma towards sex workers, they are unlikely to actively seek HIV prevention and treatment services despite access to antiretroviral therapy (ART) being free in China as part of the government national program [61]. This study showed that there was both limited engagement and follow up with health professionals. Outreach workers need to increase contacts with FSWs and provide information on HIV, by reaching out to them where they work before their evening shifts. Providing information on HIV using ‘roadside restaurants’ and pop-ups for support, where HIV information is provided in the community, as used in rural China [62] could be potential alternatives. One participant mentioned education of the community to further sensitize others around HIV. Community education programs on HIV prevention are an effective model for increasing engagement, knowledge and attitudes towards HIV [63]. This study supports the need for further community engagement, which should in turn improve HIV awareness among FSWs and reduce HIV transmission.

FSWs preferred to use condoms, and reported that they had access to the method, but that ultimately the negotiation of condom use was beyond their control. The FSWs preferred male clients to use a condom, and those who knew their HIV status insisted on use, but the women admitted that sometimes they did not use a condom. It is important for all sex workers to have access to condoms, education, and testing can act as a barrier to HIV infections while ART for FSWs who test positive can improve their immunity to opportunistic infections [48]. Education and testing can act as a barrier to being infected with HIV while ART can lead to FSWs who test positive for HIV becoming undetectable and un-transmittable (U = U campaign), meaning even though they are HIV positive they cannot infect others as their viral load is no longer high enough [64]. Importantly, the services should be available in safe environments and sex workers should be able to access them with dignity and without harassment [48]. Furthermore, it is important for the services to target both FSWs and their male clients [53], given the limited ability of FSWs to negotiate condom use with their male partners. A study in Yunnan showed that male clients are at risk of HIV infection due to their often undisclosed drug use and numerous sexual partners (20, 65). Although most studies in the Yunnan highlight the risks FSWs pose to male clients, the clients can also expose FSWs to the risk of contracting HIV/STIs, especially those living in mining areas, [66] young ethnic minority groups who are migrants, those with low levels of education, injecting drug users, and those who inconsistently use condoms [67, 68]. The FSWs can in turn transmit HIV to their main intimate partner(s), particularly if they work in the high-risk environments [69]. A considerable number of women enter sex work due to financial difficulties [19]. Participants in this study tended to be unemployed, and their financial situation reduced their power to negotiate for the condom use. Furthermore, one participant reported that she had contracted HIV from her husband due to sharing needles when injecting drugs. Considering the difficulties of negotiating condom use, HIV pre-exposure prophylaxis (PrEP) among vulnerable FSWs is a prevention method with greater level of control. However, due to lack of awareness among FSWs, PrEP has not achieved its potential effectiveness in reducing HIV infections among FSWs [70]. The long-term effects of PrEP show some concerns around a reduction in bone density [71], with women being prone to loss of bone density, which suggests that such interventions should be carefully promoted taking such risks into account. Microbicides are an alternative that FSWs have found highly acceptable to prevent HIV transmission; however, these methods are only 40% effective when compared to condoms [72]. From the experiences of FSWs in this study, there is need for interventions targeting male clients involving health promotion strategies in order to improve condom use among them.

FWSs reported that they conducted self-examination and used douching as a way to prevent transmission of HIV/STIs. Few reported having an STI but instead indicated experiencing an ‘itchy vagina’ and leucorrhea. Research has shown that one half [29] to three quarters of women who test positive for STIs are asymptomatic [30], which could explain the low reports of such infections by FSWs in this study. In general FSWs in the Yunnan area have higher rates of STIs, such as trichomonas vaginalis, and douching vaginally, all of which increase the risk of transmission of HIV (41, 73). Douching vaginally is a common practice among FSWs, which removes natural flora and pushes pathogens up through the cervix to the female reproductive system [74]. FSWs more likely to douche come from a Han background, work in low level establishments, had lower stomach pain, and work in multiple locations [75]. The practice has previously been linked to lower rates of condom use and it has historically been used by those in high risk environments to replace condoms [49]. Another study found that vaginal douching was not related to condom use but was used potentially as a method to treat STI symptoms [60]. Douching may be a common practice because of the high cost of treatment of STIs; previous research has used incentives like a 60% STI treatment discount to improve access to treatment for FSWs, which may lower vaginal douching [76]. Those with high mobility reported fewer STIs (and low HIV rates), while those who are stationed in the province and who worked in environments that were considered high risk, who saw more customers monthly, had high rates of STIs/HIV [43]. A qualitative review and meta-analysis of FSWs’ work environments found that indoor venues were safe and had better access to condoms and HIV/STI testing while outdoor venues were less safe with limited opportunity to benefit from HIV prevention [12]. One participant reported accessing gynecological services when she was engaged in indoor sex work in dance halls but not when she engaged in outdoor work. This further supports the argument that this group of FSWs were both outdoor and indoor workers who had unique needs for HIV/STI prevention. However, this complexity is rarely explored and research instead categorizes FSWs as engaging in one type of work or the other, not both. The findings from this study support previous research that suggests a need for the Chinese government to address the persistent problem of low access to HIV/STI testing among the FSWs as part of their public health campaigns, including the need to promote protecting one’s health and that HIV can be asymptomatic [77]. Additionally, this study suggests a need to include education on vaginal douching within public health campaigns and to promote vaginal health as part of programs for FSWs. Without interventions to increase vaginal health, FSWs in this study setting continue to be at a high risk of contracting HIV/STIs and infecting their male clients who could then infect the general population.

In this study few workers reported taking alcohol or using illicit drugs while working, which is in contrast to findings from a previous study which showed that more than one third of FSWs were using drugs [64]. This could be due to location as this study was conducted in the area where China meets Vietnam, and the other study was conducted in the China-Myanmar area where there could be more alcohol consumption and use of drugs. This suggests that there may be nuanced drug use patterns within the counties of the “Golden Triangle” region that warrant further investigation, as some areas may be more prone to use of drugs than others. Studies conducted in the Golden Triangle region since 2005 show that FSWs who use drugs are more likely to have HIV than those who do not (29, 37, 78). Research largely focuses on male clients and does not focus on the transmission risk between FSWs who use drugs and their male partners [79], with one study showing there is sharing of needles between the FSWs and male clients [46]. The findings of this study show that the FSWs who used drugs also talked about being infected with HIV by their male partners who also used drugs. Participants in this study, who self-identified as IDUs, discussed engaging in risky behaviors. Evidence suggests that harm reduction strategies targeting drug use are important for reducing HIV transmission in the Yunnan [5]. Needle and pip sharing are the main risky behaviors among FSWs using drugs in the region [32]. A key finding of this study is that sharing water is a risk factor for HIV infection among FSWs in the study setting. This is because it leads to sharing of blood contaminated water; injectors often rinse the syringe right after use with blood still in the syringe, thereby increasing the risk of indirect transmission. Furthermore, if a syringe is to be reused it should be washed with bleach and water [80]. Evidence shows that reuse of a syringe causes further damage to the body’s injecting sites, since after the first injection the needle immediately becomes blunt [81]. The findings suggest a need for interventions to reduce harm associated with sharing needles and water among FSWs in the study setting. The finding is consistent with that by Hail-Jares [82], which suggests that harm reduction programs are only effective with FSWs in the Yunnan if they go beyond basic care (i.e. providing clean needles, methadone) to provide advice such as what gauge of needle to use depending on injection site, not to share water, and use a syringe only once. Drug use is associated with low likelihood of condom use and high likelihood of HIV infection among FSWs [83]. However, as this study has shown, most FSWs reported working when sober or with a maximum of two drinks, which suggests that the participants were engaging in less risky behavior than those in other studies. Few FSWs reported drug use in this study, which could partly be due to discomfort in disclosing such behavior, which suggests a need for further research on the topic. One way to address risks around drug use and HIV is to integrate sexual and reproductive health services into drug services for FSWs [84]. Integrated services would eliminate the need to form more than one trusting relationship with providers, which is required when attending both sexual and reproductive health services and harm reduction services separately.

The study has certain limitations. First, Yunnan is a special geographic region for studying HIV because it has the highest rate of HIV infection through heterosexual intercourse, and the findings may not represent the general situation of FSWs in China. Second, the study used a convenience sample where researchers interviewed sex workers who were easy to identify and contact. However, the more isolated FSWs (e.g., those controlled by pimps) could be at greater risk of HIV and should be considered in future studies. Third, the study did not consider the views of male clients of FSWs whose perspectives and experiences could be different from those of FSWs who participated in the study. We recommended future research could focus on both FSWs and their clients to explore the complexity of HIV related risks. Fourth, this study did not capture the experience of transgendered FSWs, who face greater HIV/STI risks than heterosexual FSWs. Fifth, we did not examine the experiences of pregnant FSWs, and could not therefore determine if they face additional HIV/STI risks than other FSWs. Sixth, besides HIV-related risk, FSWs also face occupational risks including physical and verbal assaults, something this study did not focus on. Experiences of sex workers who used gynecological services but did not report having any STI symptoms were not captured, which could have provided important insights into the drivers of care-seeking for gynecological conditions among FSWs.

## Conclusions

In conclusion, the finding that there is low HIV awareness and low use of gynecological services suggests a need for new interventions for HIV prevention among FSWs and their clients. It is essential for governmental agencies to implement practical strategies in terms of both HIV prevention and condom use for FSWs in China, such as raising awareness around the increased risks of HIV/STIs from douching. The prevalence of risky behaviors among certain groups of FSWs suggests a need for interventions targeting the most vulnerable segments, including older women, those with limited ability to negotiate condom use, those with limited access to gynecological and HIV support services, and those injecting drugs or taking alcohol. In order to adequately reach such groups, interventions should employ innovative strategies such as reaching out to FSWs where they work, and in the community, adapting something like a ‘roadside restaurant’ (a hotel in the same area as an indoor venue where sex work occurs), which has been successful in rural China. The findings of the study further suggest that mobile outreach is another effective strategy for reaching FSWs with preventive information and services.

## Supplementary Information

**Additional file 1:** Semi-structured interview topic schedule.

## Data Availability

The data used in this paper are not publicly available due to the highly sensitive nature of the data, but are available from the corresponding author on reasonable request. The topic schedule has been made available in Additional file 1.

## Abbreviations

FSWs: Female sex workers
HIV: Human immunodeficiency virus
AIDS: Acquired immune deficiency syndrome
PWIDs: People who inject drugs
STIs: Sexually transmitted infections
IDU: Injecting drug use
ART: Antireviral treatment
PrEP: Pre-exposure prophylaxis

## References

[1] Baral S, Beyrer C, Muessig K, Poteat T, Wirtz AL, Decker MR, Sherman SG, Kerrigan D (2012). Burden of HIV among female sex workers in low-income and middle-income countries: a systematic review and meta-analysis. Lancet Infect Dis.

[2] Sutherland D, Hsu JY (2013). HIV/AIDS in China-the economic and social determinants.

[3] Zhang H, Hsieh E, Wang L, Liao S (2020). HIV/AIDS among female sex workers in China: epidemiology and recent prevention strategies. Curr HIV/AIDS Rep.

[4] Zeng H, Zhang L, Zhao Y, Liu H, Guo H, Wang Y, Zhang Z, Mao L (2016). HIV prevention among street-based sex workers (SSW s) in Chongqing, China: interviews with SSWs, clients and healthcare providers. Health Soc Care Community.

[5] Hong Y, Poon AN, Zhang C (2011). HIV/STI prevention interventions targeting FSWs in China: a systematic literature review. AIDS Care.

[6] Wu X, Huang H, Tang Z, Shen Z, Lu H, Chen H, Chen L, Huang H, Ruan Y, Shao Y (2017). Aphrodisiac use and associated factors among older male clients of low-cost female sex workers in Southwestern rural areas of China. Sex Res Soc Policy.

[7] Chen H, Wu X, Chen L, Lu H, Tang Z, Shen Z, Pan SW, Ruan Y, Shao Y (2019). Rapidly spreading human immunodeficiency virus epidemic among older males and associated factors: a large-scale prospective cohort study in rural Southwest China. Sex Transm Dis.

[8] Hong Y, Li X (2008). Behavioral studies of female sex workers in China: a literature review and recommendation for future research. AIDS Behav.

[9] Beyrer C, Crago AL, Bekker LG, Butler J, Shannon K, Kerrigan D, Decker MR, Baral SD, Poteat T, Wirtz AL, Weir BW (2015). An action agenda for HIV and sex workers. Lancet.

[10] Wilson D (2015). HIV programs for sex workers: lessons and challenges for developing and delivering programs. PLoS Med.

[11] Bekker LG, Johnson L, Cowan F, Overs C, Besada D, Hillier S, Cates W (2015). Combination HIV prevention for female sex workers: what is the evidence?. Lancet.

[12] Goldenberg SM, Duff P, Krusi A (2015). Work environments and HIV prevention: a qualitative review and meta-synthesis of sex worker narratives. BMC Public Health.

[13] Zhang XD, Myers S, Yang HJ, Li Y, Li JH, Luo W, Luchters S (2016). Prevalence and correlates of sexual and gender-based violence against Chinese adolescent women who are involved in commercial sex: a cross-sectional study. BMJ Open.

[14] Hesketh T, Zhang J, Qiang DJ (2005). HIV knowledge and risk behaviour of female sex workers in Yunnan Province, China: potential as bridging groups to the general population. AIDS Care.

[15] Chen M, Ma Y, Chen H, Dai J, Luo H, Yang C, Dong L, Jin X, Yang M, Yang L, Song L (2019). Spatial clusters of HIV-1 genotypes in a recently infected population in Yunnan, China. BMC Infect Dis.

[16] Wang J, Ding G, Zhu Z, Zhou C, Wang N (2015). Analysis of HIV correlated factors in Chinese and Vietnamese female sex workers in Hekou, Yunnan Province, a Chinese Border Region. PLoS ONE.

[17] Poshyachinda V (1993). Drug injecting and HIV infection among the population of drug abusers in Asia. Bull Narc.

[18] Xiao Y, Kristensen S, Sun J, Lu L, Vermund SH (2007). Expansion of HIV/AIDS in China: lessons from Yunnan Province. Soc Sci Med.

[19] Chen M, Yang L, Ma Y, Su Y, Yang C, Luo H, Chen H, Chen L, Yan W, Shi Y, Jia M (2013). Emerging variability in HIV-1 genetics among recently infected individuals in Yunnan, China. PLoS ONE.

[20] Jia M, Luo H, Ma Y, Wang N, Smith K, Mei J, Lu R, Lu J, Fu L, Zhang Q, Wu Z (2010). The HIV epidemic in Yunnan province, China, 1989–2007. JAIDS J Acquir Immune Defic Syndr.

[21] Wang JJ, Qian YA, Fan PY, Reilly KH, Ding GW, Ning WA (2012). Estimation of population-size changes and HIV prevalence among female sex workers from 2006 to 2009 in Kaiyuan, Yunnan, China. Biomed Environ Sci.

[22] Duan S, Shen S, Bulterys M, Jia Y, Yang Y, Xiang L, Tian F, Lu L, Xiao Y, Wang M, Jia M (2010). Estimation of HIV-1 incidence among five focal populations in Dehong, Yunnan: a hard hit area along a major drug trafficking route. BMC Public Health.

[23] Yang L, Chen M, Ma Y, Luo H, Yang C, Su Y, Chen H, Shi Y, Mei J, Jia M, Lu L (2015). The changing trends of HIV-1 prevalence and incidence from sentinel surveillance of five sub-populations in Yunnan, China, 2001–2010. BMC Public Health.

[24] Zhang T, Jia M, Luo H, Zhou Y, Wang N (2011). Study on a HIV/AIDS model with application to Yunnan province, China. Appl Math Model.

[25] Lu L, Jia MH, Lu JY, Luo H, Zhang X (2005). Analysis of HIV/AIDS prevalence in Yunnan province. J China AIDS/STD.

[26] Chen M, Ma Y, Chen H, Dai J, Luo H, Yang C, Dong L, Jin X, Yang M, Yang L, Song L (2019). Demographic characteristics and spatial clusters of recent HIV-1 infections among newly diagnosed HIV-1 cases in Yunnan, China, 2015. BMC Public Health.

[27] Chen L, His JH, Wu X, Shen Z, Lu H, Chen H, Huang H, Zhang H, Ruan Y, Shao Y, Tang Z (2017). Disparities in HIV and syphilis prevalence and risk factors between older male clients with and without steady sex partners in southwestern rural China. BMC Infect Dis.

[28] Wang H, Chen RY, Ding G, Ma Y, Ma J, Jiao JH, Wu Z, Sharp GB, Wang N (2009). Prevalence and predictors of HIV infection among female sex workers in Kaiyuan City, Yunnan Province. China Int J Infect Dis.

[29] Wen-Juan MA, Jun-Jie WA, Reilly KH, Ai-Mei BI, Guo-Wei DI, Smith K, Ning WA (2010). Estimation of probability of unprotected heterosexual vaginal transmission of HIV-1 from clients to female sex workers in Kaiyuan, Yunnan Province. China Biomed Environ Sci.

[30] Reilly KH, Wang J, Zhu Z, Li S, Yang T, Ding G, Qian HZ, Kissinger P, Wang N (2012). HIV and associated risk factors among male clients of female sex workers in a Chinese border region. Sex Transm Dis.

[31] Zhu J, Hu D, Yin Y, Zhu Z, Wang N, Wang B (2019). HIV prevalence and correlated factors among male clients of female sex workers in a border region of China. PLoS ONE.

[32] Yu HF, An XJ, Tong JY (2009). Survey of constraints and obstacles for seeking for and receiving VCT services among high risk population in Yunnan province. Chin J AIDS STD.

[33] Chen Y, Pang L, Rou K (2009). An evaluation of coverage of interventions for HIV/AIDS-related high-risk behaviors among street-based female sex workers in two counties of Yunnan province. Chin J AIDS STD.

[34] Yang JF, Hu AY, Zhao CZ (2012). Data analysis of HIV prevalence and related behaviors among female sex workers in Baoshan of Yunnan Province from 2007 to 2011. Chin J AIDS STD.

[35] Yang X, He CY, Liu XM (2009). Medical care seeking of patients with sexually transmitted infection among female sexual-workers in Yunnan province. Chin J Public Health.

[36] Wang H, Chen RY, Ding G, Ma Y, Ma J, Jiao JH, Wu Z, Sharp GB, Wang N (2009). Prevalence and predictors of HIV infection among female sex workers in Kaiyuan City, Yunnan Province, China. Int J Infect Dis.

[37] Chen XS, Yin YP, Liang GJ, Gong XD, Li HS, Shi MQ, Yu YH (2006). Co-infection with genital gonorrhoea and genital chlamydia in female sex workers in Yunnan, China. Int J STD AIDS.

[38] Chen XS, Yin YP, Liang GJ, Gong XD, Li HS, Poumerol G, Thuy N, Shi MQ, Yu YH (2005). Sexually transmitted infections among female sex workers in Yunnan, China. AIDS Patient Care STDs.

[39] Ngo TD, Laeyendecker O, Li C, Tai H, Cui M, Lai S, Quinn TC (2008). Herpes simplex virus type 2 infection among commercial sex workers in Kunming, Yunnan Province, China. Int J STD AIDS.

[40] Wang JJ, Zhu ZB, Xi YA, Jing WU, Wang HB, Lin FE, Ding GW, Norris JL, Ning WA (2012). Herpes simplex virus type 2 risks in female sex workers in the China-Vietnam border county of Hekou. Biomed Environ Sci.

[41] Luo L, Reilly KH, Xu JJ, Wang GX, Ding GW, Wang N, Wang HB (2016). Prevalence and correlates of *Trichomonas vaginalis* infection among female sex workers in a city in Yunnan Province, China. Int J STD AIDS.

[42] Korenromp EL, Zhang W, Zhang X, Ma Y, Jia M, Luo H, Guo Y, Zhang X, Gong X, Chen F, Li J (2020). the Spectrum-Sti Groups model: syphilis prevalence trends across high-risk and lower-risk populations in Yunnan, China. Sci Rep.

[43] Wang HB, Smith K, Brown KS, Wang GX, Chang DF, Xu JJ, Ding GW, Jin X, Reilly KH, Wang N (2011). Prevalence, incidence, and persistence of syphilis infection in female sex workers in a Chinese province. Epidemiol Infect.

[44] Wang HB, Wang N, Ding GW (2007). Prevalence and risk factors associated with HIV infection among female sex workers in a county of Yunnan province. Chin J AIDS STD..

[45] Li T, Tang S, Su Y, Bao Z, Wang X, Liu Y, Li H, Han J, Pei Z, Wan Z, Fan H (2020). High prevalence and viremia of human pegivirus 2 in the HIV-infected population in Honghe Prefecture, Yunnan Province. Arch Virol.

[46] Yang X, He CY, Han CL, Shi Y, Yang YQ, Shi CB, Jia CH (2009). Study on prevalence of sexually transmitted infections among female sex workers in part areas of Yunnan. Soft Sci Health.

[47] Zhou YH, Yao ZH, Liu FL, Li H, Jiang L, Zhu JW, Zheng YT (2012). High prevalence of HIV, HCV, HBV and co-infection and associated risk factors among injecting drug users in Yunnan province, China. PLoS ONE.

[48] Zhang T, Zhou Y (2016). Mathematical model of transmission dynamics of human immune-deficiency virus: a case study for Yunnan, China. Appl Math Model.

[49] Xu J, Smith MK, Ding G, Chu J, Wang H, Li Q, Chang D, Wang G, Shang H, Jiang Y, Wang N (2013). Drug use and sex work: competing risk factors for newly acquired HIV in Yunnan, China. PLoS ONE.

[50] Zhang Z, Song L, Luo H, Mei J, Lu R, Xiao M, Jia M (2014). An analysis of high risk behaviors among female sex workers in Yunnan province in 2013. Chin J Prevent Med.

[51] Yao Y, Yang F, Chu J, Siame G, Lim HJ, Jin X, Ding G, Sun Y, Wang G, Yu Y, Wang N (2012). Associations between drug use and risk behaviours for HIV and sexually transmitted infections among female sex workers in Yunnan, China. Int J STD AIDS.

[52] Wang H, Wang N, Jia M, Bi A, Wang G, Ding G, Lin L, Smith K (2009). Sexually transmitted infections among female sex workers in Kaiyuan city, Yunnan province, China: potential for HIV transmission. Sex Transm Infect.

[53] Luo X, Duan S, Duan Q, Pu Y, Yang Y, Ding Y, Gao M, He N (2013). Alcohol use and subsequent sex among HIV-infected patients in an ethnic minority area of Yunnan Province, China. PLoS ONE.

[54] Yao Y, Wang N, Chu J, Ding G, Jin X, Sun Y, Wang G, Xu J, Smith K (2009). Sexual behavior and risks for HIV infection and transmission among male injecting drug users in Yunnan, China. Int J Infect Dis.

[55] Fu Z, He N, Duan S, Jiang Q, Ye R, Pu Y, Zhao G, Huang ZJ, Wong FY (2011). HIV infection, sexual behaviors, sexual networks, and drug use among rural residents in Yunnan Province, China. AIDS Behav.

[56] Yu YJ. The moral code of chinese sex workers. sapiens anthropology magazine. viewed, 6 Oct 2020. https://www.sapiens.org/culture/china-sex-trade-moral-code/.

[57] Al-Amer R, Ramjan L, Glew P, Darwish M, Salamonson Y (2015). Translation of interviews from a source language to a target language: examining issues in cross-cultural health care research. J Clin Nurs.

[58] Braun V, Clarke V (2006). Using thematic analysis in psychology. Qual Res Psychol.

[59] Denzin NK, Denzin NK (1978). Triangulation: a case for methodological evaluation and combination. Sociological methods: a sourcebook.

[60] Zhang ZY, Song LJ, Mei JY (2015). Awareness and high risk behaviors of HIV infection among female sex workers in Yunnan border region. Chin J Public Health.

[61] Chen M, Jia MH, Ma YL, Luo HB, Chen HC, Yang CJ, Dai J, Yang L, Dong LJ, Lu R, Song LJ (2018). The changing HIV-1 genetic characteristics and transmitted drug resistance among recently infected population in Yunnan, China. Epidemiol Infect.

[62] Liao SS, He QY, Choi KH, Hudes ES, Liao JF, Wang XC, Liu M, Pan WL, Mandel JS (2006). Working to prevent HIV/STIs among women in the sex industry in a rural town of Hainan, China. AIDS Behav.

[63] Salam RA, Haroon S, Ahmed HH, Das JK, Bhutta ZA (2014). Impact of community-based interventions on HIV knowledge, attitudes, and transmission. Infect Dis Poverty.

[64] Eisinger RW, Dieffenbach CW, Fauci AS (2019). HIV viral load and transmissibility of HIV infection: undetectable equals untransmittable. JAMA.

[65] Jin X, Smith K, Chen RY, Ding G, Yao Y, Wang H (2010). HIV prevalence and risk behaviors among male clients of female sex workers in Yunnan, China. J Acquir Immune Defic Syndromes.

[66] Zhang GL, Wang N, Pu Y, Xu J, Li B, Jia M, Wang H, Zhen X, Wu Z (2007). Study on HIV/STIs related knowledge, behavior and prevalence among clients of female sex workers in a mining area of Yunnan province. Chin J AIDS STD.

[67] Zhang G, Wang N, Wong M, Yi P, Xu J, Li B, Ding G, Ma Y, Wang H, Zheng X, Wu Z (2010). HIV-1 and STIs prevalence and risk factors of miners in mining districts of Yunnan, China. J Acq Immune Defic Syndr.

[68] Xu J, Gu J, Duo L (2008). Community-based survey of HIV/STD related risk behaviors among clients of FSWs in Kaiyuan City. Chin J AIDS STD.

[69] Li YC, Li J, Jiao F (2012). Unprotected sex behavior among female sex workers in low class establishments and related factors in a city of Yunnan province. Chin J AIDS STD.

[70] Poon AN, Han L, Li Z, Zhou C, Li Y, Huang L, Liao M, Shepard C, Bulterys M (2019). Acceptability and willingness of HIV pre-exposure prophylaxis amongst female sex workers in China. AIDS Care.

[71] Tumarkin E, Siedner MJ, Bogoch II (2019). HIV pre-exposure prophylaxis (PrEP). BMJ.

[72] Wang Y, Jiang Y, Lu L, Wang G, Bi A, Fang H, Chang D, Gu J, Wang W (2010). Microbicide acceptability and associated factors among female sex workers and male clients in Kaiyuan county, Yunnan province, China. JAIDS J Acquir Immune Defic Syndr.

[73] Luo L, Xu JJ, Wang GX, Ding GW, Wang N, Wang HB (2016). Vaginal douching and association with sexually transmitted infections among female sex workers in a prefecture of Yunnan Province, China. Int J STD AIDS.

[74] Wang H, Wang N, Chen RY, Sharp GB, Ma Y, Wang G, Ding G, Wu Z (2008). Prevalence and predictors of herpes simplex virus type 2 infection among female sex workers in Yunnan Province, China. Int J STD AIDS.

[75] Wang HB, Wang N, Ma JG, Wang GX, Chang DF, Ding GW, Xu JJ, Zhang GL, Dong RL, Zhang L, Wu ZL (2007). Study on the association between vaginal douching and sexually transmitted diseases among female sex workers in a county of Yunnan province. Zhonghua Liu Xing Bing Xue Za Zhi.

[76] Su Y, Ding G, Reilly KH, Norris JL, Liu H, Li Z, Wang G, Fang G, Wang N (2016). Loss to follow-up and HIV incidence in female sex workers in Kaiyuan, Yunnan Province China: a nine year longitudinal study. BMC Infect Dis.

[77] Xu J, Brown K, Ding G, Wang H, Zhang G, Reilly K, Li Q, Wang G, Wang N (2011). Factors associated with HIV testing history and HIV-test result follow-up among female sex workers in two cities in Yunnan, China. Sex Transm Dis.

[78] Zhu J, Yuan R, Hu D, Zhu Z, Wang N, Wang B (2018). HIV prevalence and correlated factors of female sex workers and male clients in a border region of Yunnan Province, China. Int J STD AIDS.

[79] Xu JJ, Smith MK, Chu J, Ding GW, Chang DF, Sharp GB, Qian HZ, Lu L, Bi AM, Wang N (2012). Dynamics of the HIV epidemic in southern China: sexual and drug-using behaviours among female sex workers and male clients in Yunnan. Int J STD AIDS.

[80] Koester S, Booth R, Wiebel W (1990). The risk of HIV transmission from sharing water, drug mixing containers and cotton filters among intravenous drug users. Int J Drug Policy.

[81] Harris M, Rhodes T (2012). Venous access and care: harnessing pragmatics in harm reduction for people who inject drugs. Addiction.

[82] Hail-Jares K, Choi S, Duo L, Luo Z, Huang ZJ (2016). Occupational and demographic factors associated with drug use among female sex workers at the China-Myanmar border. Drug Alcohol Depend.

[83] Wu P, Dong WM, Rou K, Dong W, Zhou C, Chen X, Zheng J, Scott SR, Wu Z (2019). HIV-positive clients of female sex workers in Hunan Province, China: a mixed methods study assessing sexual relationships and risk behavior by type of partner. BMC Public Health.

[84] Zhang XD, Kelly-Hanku A, Chai JJ, Luo J, Temmerman M, Luchters S (2015). Sexual and reproductive health risks amongst female adolescents who use amphetamine-type stimulants and sell sex: a qualitative inquiry in Yunnan, China. Harm Reduct J.

