# Supplementary material for: A thematic analysis of experiences of HIV risks among female sex workers in the Yunnan-Vietnam Chinese border region
Source: BMC Womens Health. 2021 Jan 6;21:7. doi: 10.1186/s12905-020-01143-x (PMC7789185; doi:10.1186/s12905-020-01143-x)
Supplement: Supplementary file 1 — Additional file 1: Semi-structured interview topic schedule. [file 12905_2020_1143_MOESM1_ESM.docx]

**Additional File 1. Semi-Structured Interview Topic Schedule**

Ice Breakers:

Tell me something about yourself…

How long have you worked as a sex worker?

Domain 1: Current Situation

Where do you work?

Tell me about what it is like to work there…

Do you have anyone you work with?

What type of work do you do?

How do you prepare before going out?

Do you take alcohol or use drugs?

Follow up: If so (for alcohol), what do you typically drink? How much? If so (for drugs), what drugs do you use? How much?

When working do you take alcohol or use drugs?

Tell me whatever you are comfortable with about your clients, keeping in mind not to provide any identifying information.

Probes- Where do you meet them? How would you describe their demeanour? Do they take alcohol or use drugs? What number of clients do you see during a typical work shift?

Do you feel you can negotiate condom use?

Follow up: If so, how important is it to you? Who asks to use a condom? How often are condoms used by clients? Is there a situation where a client would not use a condom? If you cannot negotiate condom use, why do you think that is?

What do you charge clients and for what?

Have you ever had any gynaecological diseases?

How long do you think you may work as a sex worker?

Domain 2: Reasons for Sex Work

Why did you start sex work?

What do you spend your wages on that you earn?

How do you find the work schedule?

Probes- Is it flexible working? How many days do you work? How many hours?

Do you enjoy your work?

Follow up: How would you describe your stress and wellbeing?

Do you have anyone you know who does sex work? Remember not to share any names or identifying information about that person/ people.

Probes- Family? Friends?

Domain 3: Border Flow Pattern

Since you work in the boarder of China and Vietnam, which counties do you work in?

Follow up: Why do you work in the county/countries?

If they work in both countries,

How often do you work in each country?

How long do you spend in each country?

How easy is it to cross the border for work?

Is there anything specific to each country that leads you to work there?

Probe- Difference? Similarities?

Where do your clients come from? Remember not to provide any detailed information that could identify them, you could for example say China, Vietnam, Abroad.

Domain 4: HIV Transmission

Tell me, what do you know about HIV/AIDS?

How do you think HIV is transmitted?

If you wanted information on HIV/AIDS where would you go?

How would you describe your physical health?

How would you describe your vaginal health?

What can be done to prevent STIs?

What can be done to prevent HIV?

Do you get a sexual health check?

Follow up: If so, how often? Why do you seek a health check? If not, why not? What could be changed for you to get a sexual health check?

Have you ever had an HIV test?

Follow up: If so, when was your last HIV test? How often do you test? Do you know your HIV status? If not, why?

How would you know if HIV is transmitted?

Do you worry that some clients have something wrong with them, like an STI or HIV/AIDS?

Follow up: Can you tell if a client has something wrong? If so how? If not, why not?

*Ending:* That is all the questions I have. Is there anything else we did not discuss that you would like to tell me?

Thank you for participating, this is the end of the interview.
